# Bio-Inspired Silver Nanoparticles Impose Metabolic and Epigenetic Toxicity to *Saccharomyces cerevisiae*

**DOI:** 10.3389/fphar.2019.01016

**Published:** 2019-09-12

**Authors:** Piyoosh Kumar Babele, Ashwani Kumar Singh, Amit Srivastava

**Affiliations:** ^1^Department of Biological Sciences, Indian Institute of Science Education and Research Bhopal, Bhopal, India; ^2^School of Physical Sciences, Jawaharlal Nehru University, New Delhi, India; ^3^Department of Physics, TDPG College, VBS Purvanchal University, Jaunpur, India

**Keywords:** silver nanoparticles (AgNPs), nanotoxicity, ^1^H-NMR, metabolomics, histones, *Saccharomyces cerevisiae*

## Abstract

Silver nanoparticles (AgNPs) have many applications in various fields, including biomedical applications. Due to the broad range of applications, they are considered as the leading fraction of manufactured nanoparticles. AgNPs are synthesized by different types of chemical and biological (green) methods. Previously, biologically synthesized AgNPs were considered safe for the environment and humans. However, new toxicity evidence have initiated a more careful assessment to delineate the toxicity mechanisms associated with these nanoparticles. This study demonstrates the use of aqueous gooseberry extract for AgNP preparation in a time- and cost-effective way. Ultraviolet-visible spectroscopy, X-ray diffraction, transmission electron microscopy, and dynamic light scattering confirm the formation of AgNPs, with an average size between 50 and 100 nm. Untargeted ^1^H-nuclear magnetic resonance-based metabolomics revealed manyfold up- and down-regulation in the concentration of 55 different classes of annotated metabolites in AgNP-exposed yeast *Saccharomyces cerevisiae* cells. Based on their chemical nature and cellular functions, these metabolites are classified into amino acids, glycolysis and the tricarboxylic acid (TCA) cycle, organic acids, nucleotide metabolism, urea cycle, and lipid metabolism. Transcriptome analysis revealed that the genes involved in oxidative stress mitigation maintain their expression levels, whereas the genes of the TCA cycle and lipid metabolism show drastic down-regulation upon AgNP exposure. Moreover, they can induce alteration in histone epigenetic marks by altering the methylation and acetylation of selected histone H3 and H4 proteins. Altogether, we conclude that the selected dose of biologically synthesized AgNPs impose toxicity by modulating the transcriptome, epigenome, and metabolome of eukaryotic cells, which eventually cause disequilibrium in cellular metabolism leading to toxicity.

## Introduction

The incorporation of engineered nanomaterials (ENMs) in several commercial applications and consumer products has been increasing rapidly. The accelerating and unregulated release of ENMs into the environment constitutes an emerging and widespread form of pollution (Oberdörster et al., 2005; Musee, 2011). Among the wide range of ENMs, silver nanoparticles (AgNPs), in particular, are considered as a major fraction of such nanoparticles. They exhibit antimicrobial and antitumor properties, leading to their application in several aspects of biomedical fields. They are often used in wound dressings, surgical instruments, medical catheters, and bone prostheses as well as in nanotheranostics and personalized medicine (Smolkova et al., 2017; Marassi et al., 2018). Earlier, biologically (using plant and microbial cell extracts) synthesized AgNPs were considered to be nontoxic for humans and its exposure with overdose was the only known parameter reported to impose toxicity (McShan et al., 2014; Singh et al., 2014; Vazquez-Muñoz et al., 2017; Siddiqi et al., 2018). However, in the recent past, several new toxicity evidence have initiated a more careful assessment to outline the toxicity mechanism(s) of AgNPs on various living systems as reviewed elsewhere (McShan et al., 2014; Vazquez-Muñoz et al., 2017). The literature describes that these mechanism(s) involve mechanical and physical damages to cell membranes by direct attachment resulting in the disruption of membrane potential. Among them, the release of silver ions (Ag^+^) and the generation of reactive oxygen species (ROS; oxidative stress) are assumed as the main factors closely associated with inflammatory cell responses, immunotoxicity, DNA damage, and global gene and/or protein expression and thus AgNP-induced toxicity (Dubey et al., 2015; Mytych et al., 2017).

Concrete information on AgNP-induced toxicity biomarkers is still lacking. Therefore, researchers are attempting to employ different “-omics”-based tools to gain a better understanding of the toxicity mechanism and precise biomarkers (Bouhifd et al., 2013). In this context, untargeted metabolomics offers a powerful tool for the quantification of small metabolites (Schnackenberg et al., 2012). Compared to transcriptomic and proteomic data, assessing the cellular metabolic alteration with nuclear magnetic resonance (NMR)-based metabolomics provides more valuable information for enlightening unknown biological effects and helps in better understanding the mechanisms of nanomaterial-induced toxicity (Lv et al., 2015). Researchers recently used this method to identify and characterize deregulated metabolites in various model systems under different physical and chemical stressors, including nanomaterials, but none has been used to assess the changes in most commonly used AgNP exposure in yeast *Saccharomyces cerevisiae* yet (Carrola et al., 2016; Carrola et al., 2018). Moreover, previous studies on nanomaterials revealed that, apart from cytotoxicity, genotoxicity, and immunotoxicity, nanomaterials used in personalized medicine could alter the cellular epigenome and induce epigenetic toxicity. These alterations were found to be associated with many pathophysiological diseases, including cancer (Smolkova et al., 2015; Sierra et al., 2016; Smolkova et al., 2017). Post-translational modifications (PTMs) of histone proteins, in particular, attained much attention as they are linked to a variety of biological processes, e.g., gene/protein regulation and cell signaling, as well as to disease states (Jenuwein and Allis, 2001). In case of AgNPs, most of these studies were focused on the DNA methylation mechanism of epigenetics, but the study that specifically deals with histone PTMs has not been reported. The availability of the whole-genome sequence, mutant strains, and metabolome database (YMDB) of *S. cerevisiae* makes it an excellent model organism to study the metabolomics and other “-omic” (e.g., epigenomic) approaches under diverse external stimuli, including nanotoxicity (Pereira et al., 2018).

The present study proceeds with the green synthesis (using the aqueous extract of gooseberry) and characterization of AgNPs. Thereafter, ^1^H-NMR analysis of the metabolic alterations induced by biologically synthesized AgNPs in the yeast *S. cerevisiae* has been done. Further, transcriptomics studies have been performed to validate the down-regulation in the expression of genes associated with the deregulated metabolites of different metabolic pathways. Finally, it is aimed to examine the potential effects on histone PTMs and its relevance in gene expression. The combined results of the present study demonstrate that biologically synthesized AgNPs significantly alter the metabolic/epigenetic markers in yeast and also illustrate a novel finding on the mechanism of nanotoxicity.

## Materials and Methods

### Chemicals and Synthesis of AgNPs

Silver nitrate (AgNO_3_) procured from Alfa Aesar was used without any further purification. Dried gooseberry was purchased from a local market (New Delhi, India). Gooseberry extract was prepared by rehydrating 25 g dried powder of gooseberry in 500 mL sterile deionized (DI) water and kept for 24 h. Further, the solution was filtered (by 0.5 µm membrane filter) and tested for contamination, if any, and stored in a cool, dark place under sterile conditions. AgNPs were synthesized by mixing gooseberry extract and AgNO_3_ (0.1 M) with constant stirring at 250 rpm at room temperature. To study the effect of the amount of gooseberry extract on the synthesis of AgNPs, two samples with different volume ratios of AgNO_3_ solution and gooseberry extract (1:1 and 1:0.5) were prepared. The instant change in color depends on the shape and size of the particles, which primarily indicate the successful formation of AgNPs. Reaction mixtures thus obtained were further centrifuged at 2000× *g* for 15 min. AgNPs thus settled at the bottom of tubes and were collected and suspended in sterile DI water.

### Characterization of Synthesized AgNPs

*X-ray diffraction (XRD) analysis:* To confirm the formation of AgNPs using gooseberry extract, XRD analysis was carried out on a tabletop diffractometer (Rigaku Miniflex 600) using CuK_α_ radiation in a wide range of Bragg angle (20° ≤ 2θ ≤ 80°).

*Transmission electron microscopy (TEM) analysis:* TEM investigations were carried out to determine the size and shape of biologically synthesized AgNPs. The samples for TEM analysis were prepared by placing a drop (∼6 µL) of dispersed AgNP suspension on 300-mesh carbon-coated copper grid and allowed to dry. Studies were done on JEOL-2100F (JEOL, USA) operated at an accelerating voltage 200 kV.

*Ultraviolet (UV)-visible (UV-vis) spectroscopy:* The formation of AgNPs was observed by evaluating UV-vis spectra of synthesized AgNP nanoparticles. The synthesized samples were analyzed using Perkin-Elmer Lambda 750S UV-vis spectrometer with a resolution of 1 nm. Then, 500 µL AgNP solution, diluted to 2 mL with water, was analyzed by a UV-vis spectrometer. Two different samples of AgNPs prepared with varying AgNO_3_ solution/gooseberry extract ratios (1:1 and 1:0.5) resulted in distinct colors due to the different shapes and sizes of the synthesized nanoparticles.

### Yeast Growth and AgNP Treatment

Overnight-grown yeast *S. cerevisiae* BY4741 (MATa; his3Δ1; leu2Δ0; met15Δ0; ura3Δ0) cells inoculated into 250 mL YPD medium (OD_600 nm_ = 0.2) were used for cytotoxicity and metabolite extraction. Yeast cells were grown until mid-exponential growth phase (OD_600 nm_ = 1.3–1.5), subsequently collected by centrifugation (1000× *g*; 5 min), and thoroughly washed three times with autoclaved Milli Q water. Water-suspended yeast cells were equally partitioned (10 mL each) into five culture tubes under sterile conditions; one tube was left untreated (control), whereas the others were used for AgNP treatment. AgNP stock solution (0.5 mg mL^-1^) was prepared in sterile DI water, further ultrasonicated for 1 h, and stored in the dark at room temperature.

### Cytotoxicity, Cell Death, and ROS Measurements

Four concentrations (0.5, 1.0, 1.5, and 2.0 mg L^-1^) of AgNPs were prepared to be used for cytotoxicity, cell death, and ROS assays. For these assays, DI water-suspended yeast cells with different doses of AgNPs were incubated for 3 h (200 rpm, 30°C). Each experiment was performed in triplicate with similar culture/treatment conditions. The cytotoxicity and cell death measurements in yeast cells under AgNP exposure were analyzed by colony-forming units (CFU) and spot sensitivity assay methods, whereas cell death assay and ROS measurements were performed in accordance with our previously published research article (Babele et al., 2018). CFU and spot assay plates were scanned using an HP scanner and the respective colonies were counted and presented as percent survival (Babele et al., 2018). Propidium iodide (PI) and 2′,7′-dichlorofluorescin diacetate (DCFDA) fluorogenic probes were used for the detection of dead cells and ROS, respectively, using previously described methods (Babele et al., 2018). The percentage of PI-positive (dead) cells was calculated as the number of PI-positive cells divided by the number of total cells × 100. Approximately 300 cells from 10 different panels (under a fluorescence microscope) were counted from three independent repeats. For ROS measurement, fluorescence intensity (FLU; excitation λ = 488 nm and emission λ = 520 nm) was used (Babele et al., 2018). All fluorescence microscopic observations were done on a Zeiss Apotome 2 using 63× oil lens in RFP (PI stain)-GFP (DCF stain) filter sets. Quantitative estimation of ROS was done in a fluorescence microplate reader (BioTek FLx800, BioTek Instruments, Inc., USA) with FLU (excitation λ = 488 nm and emission λ = 520 nm).

### Metabolite Extraction and Sample Preparation for NMR Analysis

For metabolite extraction, sterile DI water-suspended yeast cells were equally divided (100 mL each) into two flasks under sterile conditions: one was left untreated (control) and the other was treated with AgNPs (1.0 mg L^-1^). The respective experiments were performed in triplicate in similar culture/treatment conditions. Metabolites were extracted according to a previously described method (Airoldi et al., 2015; Babele, 2019). Briefly, the control and treated cells were pelleted through centrifugation and washed gently with 100 mL ice-cold water and instantaneously placed in a refrigerator (-80°C). Frozen cells were thoroughly washed (three times) and rinsed with 10 mL ice-cold water. Further, cell pellets were resuspended in 4 mL of 75%:25% ethanol/DI water and an equal volume of autoclaved glass beads was subsequently added. Tubes were allowed to be vigorously vortex mixed (10 times) for 35 s with a time interval of 40 s in the ice. Further, samples were centrifuged (5 min, 2000× g) to remove the glass beads. Cell debris from the extract and equal volumes of the supernatant (with metabolites) were vacuum evaporated. Finally, dried samples were properly dissolved in 600 μL deuterated phosphate buffer (Na_2_DPO_4_ 100 mM, pH 7.5) in D_2_O. For chemical shift and the calculation of concentration, 0.1 mM 4,4-dimethyl-4-silapentane-1-sulfonic acid (DSS-d_6_) was used as an internal standard.

### ^1^H-NMR Experiments and Spectra Preprocessing

An Advance-III 700 MHz FT NMR spectrometer (Ascend^TM^, Bruker) in digital quadrature detection mode at a frequency of 4323.96 Hz 5 mm QCI cryoprobe was used to assess NMR spectra (Babele, 2019). The deconvolution of the overlapping regions of the spectra and subsequently the absolute quantification for metabolites with resonance in populated spectral areas were performed (Puig-Castellví et al., 2016).

### Metabolite Identification and Quantification

A targeted metabolite profiling analysis of ^1^H-NMR signals using both YMDB and HMDB was done for metabolite assignment. During analysis, each spectral intensity data set was normalized to the assigned chemical compounds in accordance with the total sum of the spectral regions. It was further converted to tab-delimited text (.txt) and comma-separated values (.csv) and fetched directly into Web-based MetaboAnalyst version 3.5 software to carry out data normalization, scaling, and multivariate analysis. p < 0.05 was used to provide the respective statistical significance. For the normalization of metabolite levels, log_2_ function and mean centering Pare to scaling were incorporated to all principal component analyses (PCA) and heatmaps by MetaboAnalyst version 3.5 (Xia et al., 2015).

### Isolation of Total RNA and Quantitative Real-Time PCR (qRT-PCR)

To carry out qRT-PCR experiments, different concentrations (0.5, 1.0, and 2.0 mg L^-1^) of AgNPs were prepared. DI water-suspended yeast cells with varying doses of AgNPs were incubated for 3 h (200 rpm, 30°C). Each experiment was performed in triplicate under similar culture/treatment conditions. RNA extraction, cDNA preparation, and corresponding qRT-PCR experiments were carried out according to a previously described method (Babele, 2019). qRT-PCR was performed in an ABI-7300 with Sequence Detection System version 1.4 software (Applied Biosystems, CA) using 2× SYBR Green Master Mix (ABI). Housekeeping gene actin (ACT1) was used as an internal control to calculate the relative expression of genes. Relative changes (control vs. AgNPs) were calculated using the 2^-ΔΔCT^ method and results are expressed as fold change. Data are the mean ± standard error of three independent experiments. The details of the genes and primers used in the present study for real-time PCR experiments are provided in **Table S1**.

### Preparation of Protein Extracts and Immunoblotting Analysis

To carry out Western blotting experiments, different concentrations (0.5, 1.0, and 2.0 mg L^-1^) of AgNPs were used. DI water-suspended yeast cells with different doses of as-prepared AgNPs were incubated for 3 h (200 rpm, 30°C). Each experiment was performed in triplicate under similar culture/treatment conditions. Protein cell extraction from 20% trichloroacetic acid precipitation and further immunoblotting analysis were carried out in accordance to a procedure as described in our previous article (Babele et al., 2018). Primary antibodies general H3 (Sigma, H0164), H4 (Sigma, SAB4500312) H3K4me2 (Abcam, 8895), H3K4me3 (Abcam, 32356), H3K56Ac (Sigma, SAB4200328), H4K8Ac (Abcam, 46982), and H4K16Ac (Abcam, 61240) were used to investigate global histone modifications. The same blots were re-probed with anti-Tbp, -H3, and -H4 protein and used as loading control; purified H3 and H4 proteins were used as marker to represent the correct bands (**Figure S4**). The detection of proteins was carried out using a relevant secondary antibody, IRDye^®^ 800CW goat anti-rabbit IgG or anti-mouse IgG (1:15,000; LI-COR Biosciences). An Odyssey infrared imager (LI-COR Biosciences) was used to scan the blots, and as-received images from three independent performances for each condition are shown.

### Statistical Analysis

The analyzed data are the mean ± SD of three independent experiments. It favors the analysis of variance (ANOVA) assumptions as received from Shapiro-Wilk normality and Levene test for the equality of variances. Statistical significance (p < 0.05) was assessed using one-way ANOVA using GraphPad Prism 5 with Dunnett’s multiple comparison test.

## Results

### Synthesis and Characteristics of AgNPs

To test whether gooseberry extract has potential to synthesize AgNPs, we performed routine and confirmatory experiments. Initially, XRD analysis was performed. **Figure S1A** shows a typical XRD pattern of AgNPs synthesized with different concentrations of AgNO_3_ and gooseberry extract. The positions of the diffraction peaks of both samples were found to be in accordance with their reference of AgNPs and indexed well with JCPDS number 04-0783. The formation of AgNPs was further confirmed by a careful observation of change in color for AgNO_3_ solution by adding gooseberry extract. UV-vis absorption spectra of AgNPs with the above-mentioned variation of gooseberry extract exhibit prominent peaks at 442 and 457 nm (**Figure S1B**). It also reveals that the absorption spectrum with a 1:1 concentration ratio possesses two distinct maxima at 342 and 457 nm. The maxima peak of the longer-wavelength side shifted to 442 nm with a varied concentration of 1:0.5 of AgNO_3_ and gooseberry extract. The morphology and microstructure of the synthesized AgNPs were examined by TEM. **Figure S2A** depicts the TEM micrographs of AgNPs obtained from 1:0.5 reaction mixture of AgNO_3_ and gooseberry extract and the respective selected area electron diffraction (SAED) pattern was indexed well with the known F.C.C. structure of AgNPs. Further, it was found that, as we increase the ratio of AgNO_3_ to gooseberry extract from 1:0.5 to 1:1, the size of the synthesized particles increased and was found in the range of 80 to 90 nm. This change in the size of the nanoparticles was likely assumed as the primary cause for the red shift in the absorption spectra of AgNPs (**Figure S1B**). The SAED patterns associated with **Figure S2B** display four diffraction rings that correspond to the (111), (200), (220), and (311) reflection planes and were in good agreement with the XRD results. **Figures S2C, D** reveal an approximate statistical distribution of the particle size.

### AgNPs Cause Dose-Dependent Toxicity, Cell Membrane Damage, and ROS Generation

**Figure 1:**
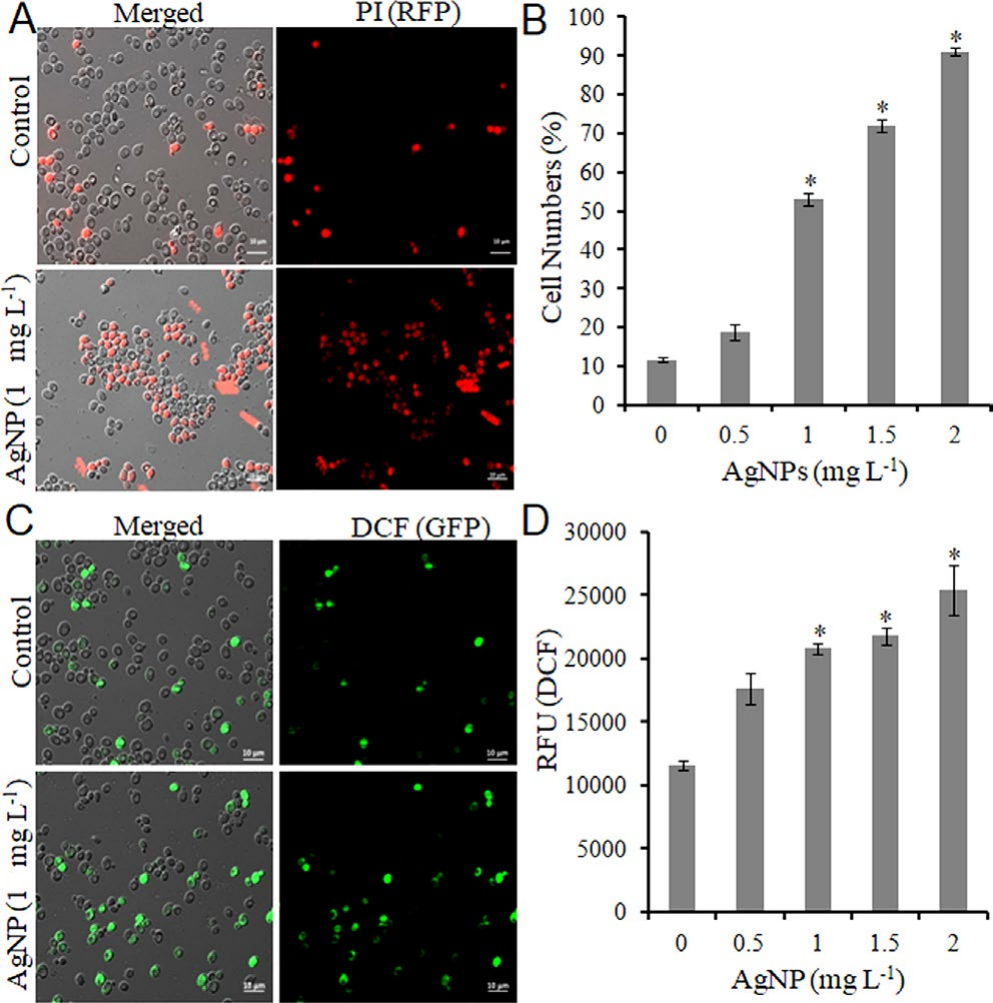
AgNPs causes cell death and oxidative stress by ROS accumulation. **(A)** Representative microscopic images of PI-stained yeast cells of control and AgNP-treated groups after 3 h. **(B)** Percentage of PI-positive (dead) cells was calculated as the number of PI-positive cells divided by the number of total cells × 100. **(C)** Representative microscopic images of DCFDA-stained yeast cells of control and AgNP-treated groups after 3 h. **(D)** ROS level in control and AgNP-treated cells was measured by a fluorescence plate reader after staining with DCFDA. Error bars, SD (n = 3). *p < 0.05, significant difference between the control and treatment groups. Bar, 10 μm.

Do biologically synthesized nanoparticles impose toxicity to biological systems? To answer this question, the effect of AgNPs on the growth of *S. cerevisiae* (BY4741) was studied. First, yeast cells were grown under different concentrations of AgNPs (0.5, 1.0, 1.5, and 2.0 mg L^-1^, respectively) to select the lethal concentration (LC_50_). A growth inhibition (CFU and spot assay) study indicated no apparent inhibition activity against cell growth with 0.5 mg L^-1^ AgNPs; however, 1.0 mg L^-1^ AgNPs led to a substantial decrease in cell numbers with 50% survival. Further, with the increase in AgNP concentration, i.e., 1.5 and 2.0 mg L^-1^, a drastic reduction in the cell numbers resulting in 20% cell survival was observed (**Figures S3A– C**). Furthermore, AgNP-exposed cells after PI staining were visualized under a microscope to investigate if the growth inhibition results in cell membrane damage. Fluorescence microscopy images (**Figure 1A**) depict that the selected doses (0.5–2.0 mg L^-1^) of AgNPs result in a gradual increase in PI-positive cells than the control cells (**Figure 1B**). Moreover, a dose-dependent gradual increase in ROS compared to control cells upon AgNP treatment was observed (**Figure 1C**). A similar trend was followed in a plate reader experiment (**Figure 1D**). These results helped us to select 1.0 mg L^-1^ as a LC_50_.

### Do AgNPs Cause Deregulation in the Metabolome and Transcriptome?

**Figure 2:**
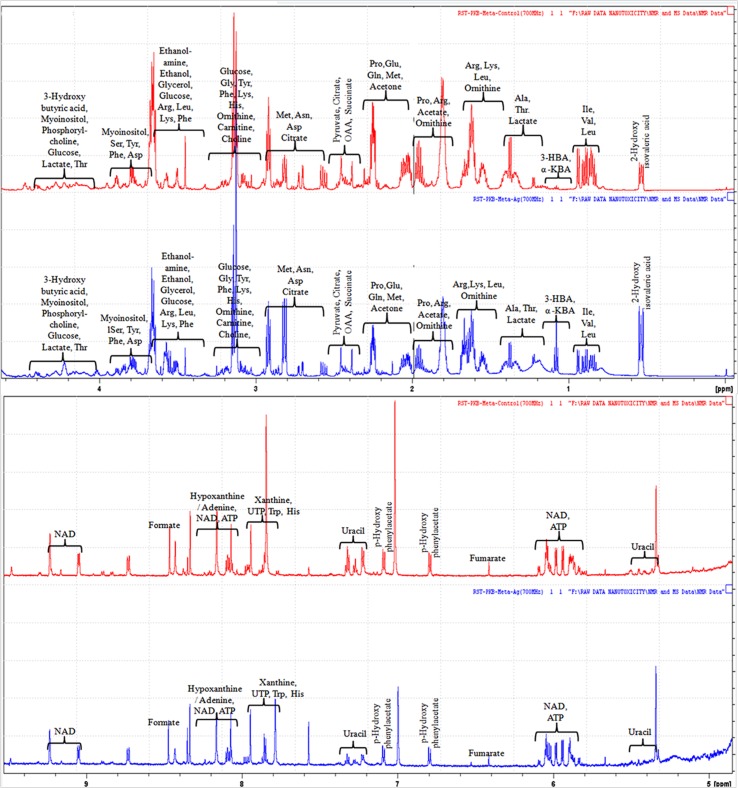
^1^H-NMR (700 MHz) spectra showing different types of metabolites, annotated based on their chemical shift (ppm) patterns. Spectra are obtained from whole-cell extracts of yeast *S. cerevisiae* cells incubated for 3 h in the absence of AgNPs (controls; red spectra) and exposed to AgNPs (1 mg L^-1^; blue spectra).

**Figure 3:**
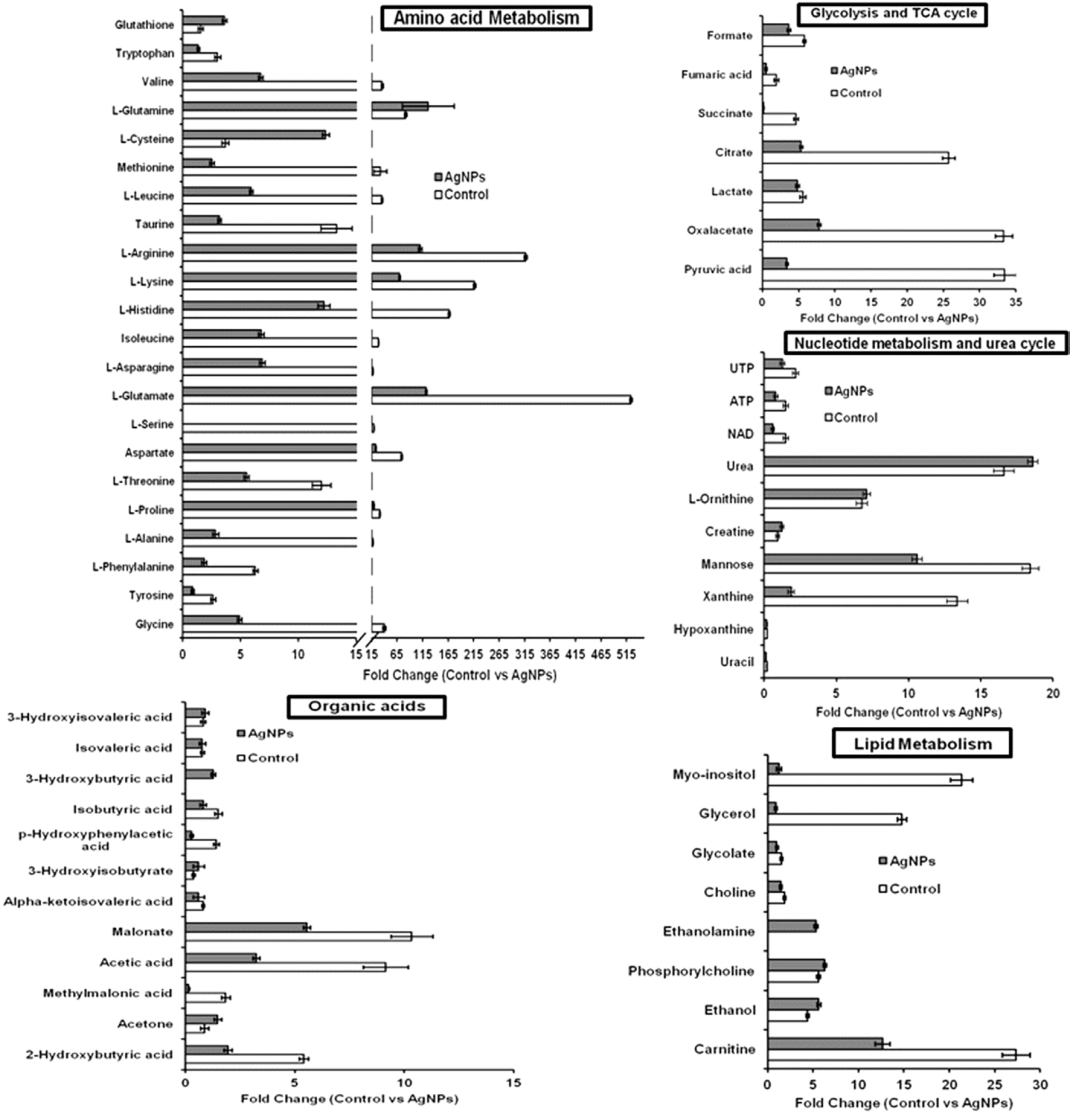
Deregulation in the metabolites involved in key metabolic pathways upon AgNP (1 mg L^-1^) exposure to *S. cerevisiae* BY4741 for 3 h. Relative changes in metabolite levels are plotted as fold change (control vs. AgNPs). Data are mean ± SE of three independent biological replicates. Values are normalized with 0.1 mM DSS-d_6_ (internal standard).

**Figure 4:**
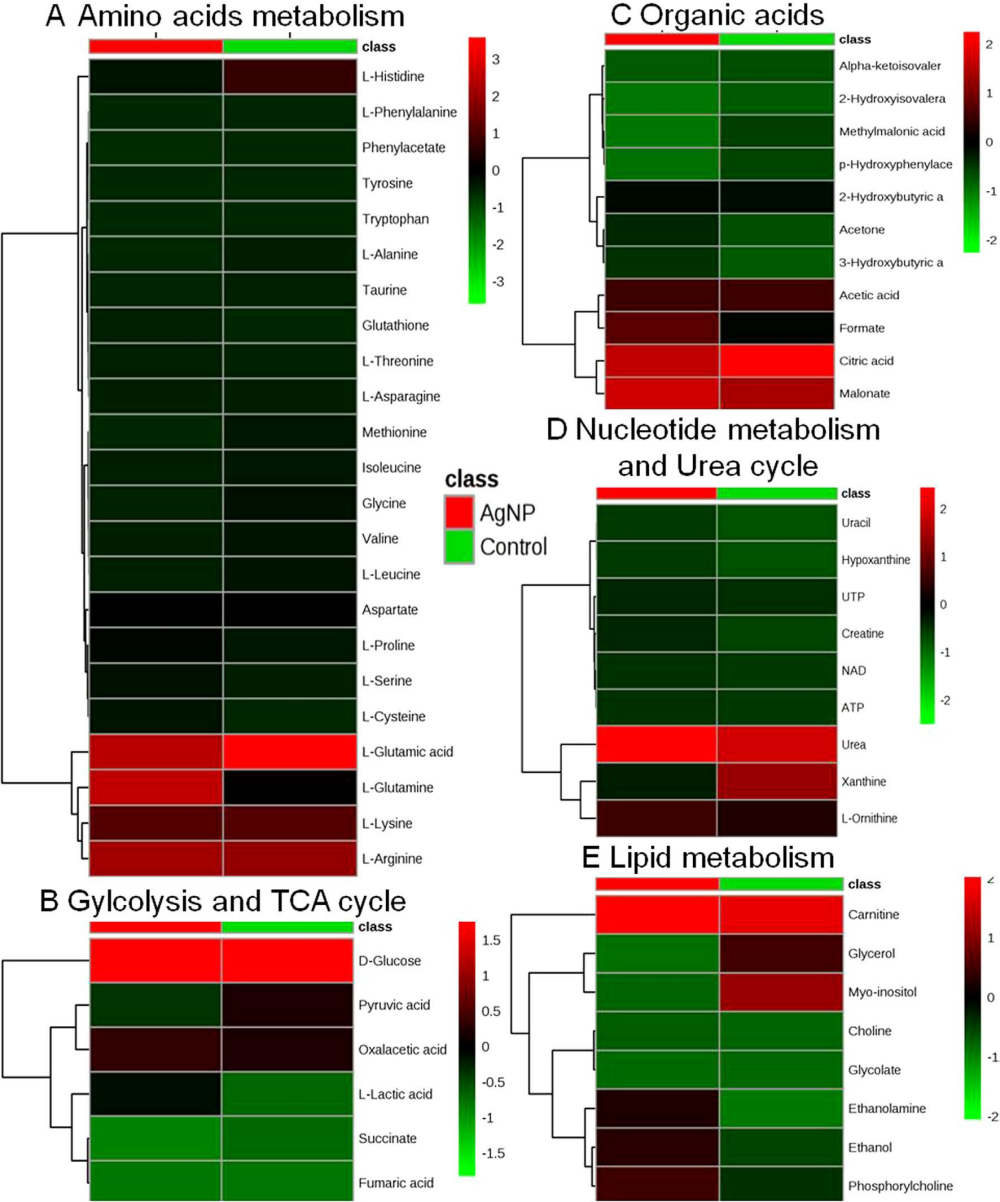
Heatmaps showing alteration in the metabolites of control vs. AgNP-treated yeast cells. The metabolites based on their chemical nature and metabolic functions are classified into **(A)** amino acid metabolism, **(B)** glycolysis and TCA cycle, **(C)** organic acids, **(D)** nucleotide metabolism and urea cycle, **(E)** and lipid metabolism.

**Figure 5:**
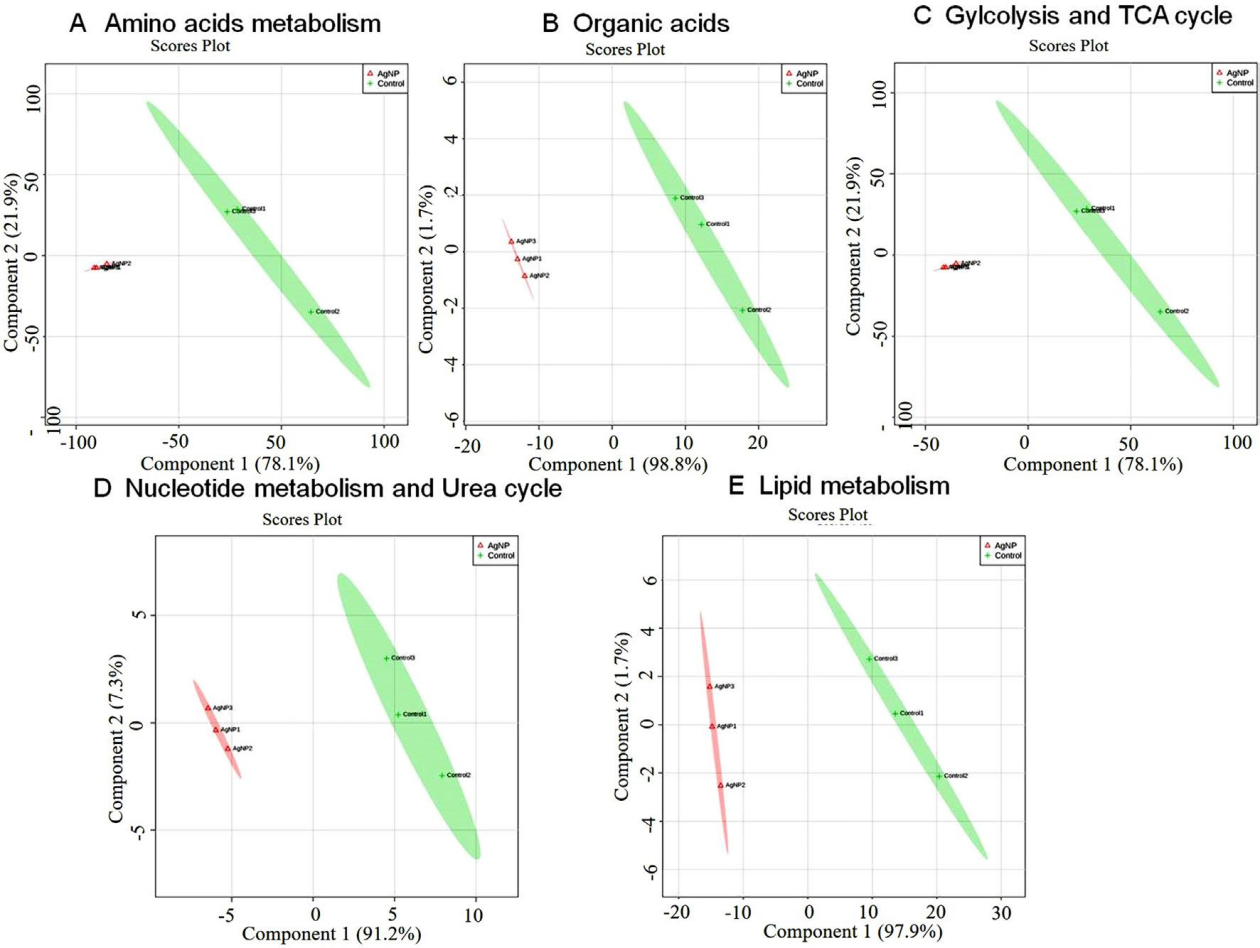
Principal component scores plot (PC1 vs. PC2) of partial least-squares discriminant analysis showing alteration in the different classes of metabolites in control vs. AgNP-treated yeast cells. The metabolites based on their chemical nature and metabolic functions are classified into **(A)** amino acid metabolism, **(B)** glycolysis and TCA cycle, **(C)** organic acids, **(D)** nucleotide metabolism, and **(E)** urea cycle and lipid metabolism.

**Figure 6:**
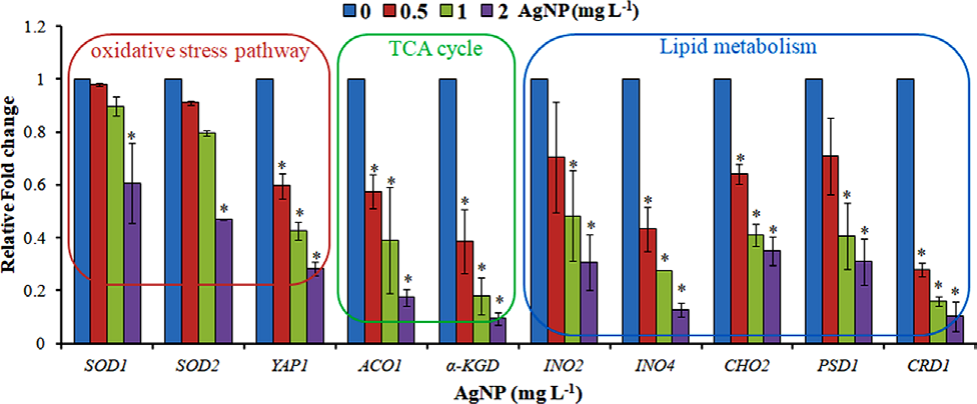
qRT-PCR showing the relative fold change in the expression profile of the genes selected from the oxidative stress pathway, TCA cycle, and lipid metabolism. Data are mean ± SE of three independent experiments. *p ≤ 0.05.

The evaluation of deregulation in metabolites was done by adopting an untargeted ^1^H-NMR analysis method. The complex experimental set-up limits the experimental conditions to a single dose of AgNPs (1.0 mg L^-1^; LC_50_). To partially overcome the complexity of metabolomic data analysis and to get insights into the molecular pathways from identified metabolite annotations, integrated metabolomic approaches were used. The respective chemical shifts resulting from ^1^H-NMR analysis have been submitted to the public database search to gain insights from the metabolite annotations and identification as described previously (Puig-Castellví et al., 2016). Based on the chemical shift data and coupling patterns, 55 metabolites were identified from *S. cerevisiae* whole-cell extracts (**Figure 2**). These annotated metabolites were classified into six different classes based on their chemical nature and metabolic functions and fold changes (control vs. AgNPs); their HMDB and KEGG IDs and cellular functions are summarized in **Table 1**. Deregulation in the metabolites were plotted in the form of fold changes (control vs. AgNP) as shown in **Figure 3**. The molecular pathway analysis conceded the fact that AgNPs exert deregulation of a wide range of key metabolites involved in amino acid metabolism, glycolysis and tricarboxylic acid (TCA) cycle (citric acid cycle), organic acids, nucleotide metabolism, urea cycle, and lipids metabolism. Results showed that AgNPs have a significant impact on the metabolic profiling of yeast cells as metabolites as shown by a significant deregulation pattern (p < 0.05). The variations in their regulation were shown in form of heatmaps (**Figure 4**). To visualize the differences in control and AgNP-treated *S. cerevisiae cells*, 55 identified metabolites were studied by PCA using MetaboAnalyst software known as a supervised clustering method to maximize the separation between groups. It is evident from the score plots that the cells divulged to AgNPs and detached to the first principal axis (PC1) and the separations between PC1 and PC2 revealed that AgNP exposure considerably altered the metabolic profiles of yeast cells (**Figure 5**). To further validate these results, some of the genes associated with these metabolic pathways have been selected. The functions of these genes are summarized in **Table S1**. Transcript-level analyses by qRT-PCR revealed that the genes of glycolysis and TCA cycle and lipid metabolism were significantly down-regulated; however, enzymes linked to oxidative stress mitigation showed the similar expression pattern in both control and treated cells (**Figure 6**).

**Table 1:** ^1^H-NMR spectroscopy (chemical shift) data and the regulation of the assigned metabolites with their respective HMDB/KEGG IDs and cellular functions.

Metabolites	Chemical shifts (ppm)	Deregulation	HMDB/KEGG ID	Cellular functions
(A) Amino acid metabolism				
Glycine	3.55 (s)	Down	0000123/C00037	Involved in glutathione and nitrogen metabolism
Tyrosine	7.18 (d); 6.89 (d); 3.97 (dd); 3.13 (dd); 3.02 (dd)	Down	0000158/C00082	Converted to NAD^+^
Phenylalanine	7.42 (m); 7.36 (m); 7.32 (d); 3.97 (dd); 3.29 (dd); 3.12 (dd)	Down	0000159/C00079	Incorporated into polypeptide chains, production of tyrosine *via* tetrahydrobiopterin-requiring phenylalanine hydroxylase and conversion to a fusel alcohol
Alanine	1.47 (d)	Down	0000161/C00041	Tightly coupled to metabolic pathways such as glycolysis, gluconeogenesis, and the TCA cycle. Also arises together with lactate and generates glucose from protein degradation *via* the alanine cycle. Alanine’s catabolic pathway directly produces pyruvate
Proline	1.90–2.12 (m); 2.27–2.40 (m); 4.12 (dd)	Down	0000162/C00148	
Threonine	4.24 (m); 1.31 (d)	Down	0000167/C00188	Yields ketogenic and glucogenic by-products
Asparagine	2.82 (d); 2.86 (d)	Down	0000168/C00152	Amino acid
Isoleucine	1.00 (d); 0.94 (t)	Down	0000172/C00407	Essential amino acid
Serine	3.94 (m); 3.83 (dd)	Down	0000187/C00065	Participates in the biosynthesis of purines and pyrimidines. Precursor to several amino acids including glycine, cysteine, and tryptophan. Also the precursor to numerous other metabolites, including sphingolipids and folate, which is the principal donor of one-carbon fragments in lipid biosynthesis
Histidine	3.11 (d); 3.15 (d); 7.06 (s); 7.81–7.92 (s)	Down	0000177/C00135	Nucleophile and a good acid/base catalyzer. Special in that its biosynthesis is inherently linked to the pathways of nucleotide formation
Lysine	3.7 (m); 3.00 (t); 1.87 (m); 1.71 (m); 1.45 (m)	Down	0000182/C00047	Important in nitrogen metabolism. Converted to acetyl CoA
Glutamate	3.74 (dd); 2.34 (td); 2.05 (m)	Down	0000148/C00025	Enters the Krebs cycle for energy metabolism and can be converted into glutamine, which is one of the key players in nitrogen metabolism
Valine	1.03 (d); 0.98 (d)	Down	0000883/C00183	Essential amino acid
Aspartate	3.88 (dd); 2.80 (dd)	Down	0000191/C00049	Precursor to several amino acids, including methionine, threonine, isoleucine, and lysine. Derived from aspartate *via* transamidation. Also a metabolite in the urea cycle and participates in gluconeogenesis. Involved in biosynthesis of inosine, the precursor to the purine bases
Cysteine	3.97 (dd); 3.06 (mm)	Up	0000574/C00097	Amino acid
L-Glutamine	2.44 (t)	Up	0000641/C00064	Plays a role in a variety of biochemical functions, including protein synthesis, cellular energy, as a source, next to glucose, nitrogen donation for many anabolic processes, and carbon donation, as a source, refilling the TCA cycle
L-Arginine	1.58–1.79 (ms); 1.80–2.00 (mm); 3.23 (t); 3.76 (t)	Down	0000517/C00062	Amino acid
Leucine	3.71 (m); 1.69 (m); 0.95 (t)	Down	0000687/C00123	Products of its breakdown are acetyl-CoA and acetoacetate. One of the two exclusively ketogenic amino acids, lysine being the other one
Tryptophan	7.7 (d); 7.5 (d); 7.3 (s) 7.1 (mm); 4.04 (dd); 3.4 (dd); 3.2 (dd)	Down	0000929/C00078	Amino acid
Taurine	3.42 (t); 3.25 (t)	Down	0000251/C00245	Derivative of the sulfur-containing amino acid cysteine and one of the few known naturally occurring sulfonic acids
Methionine	3.8 (dd); 2.63 (t); 2.12 (s)	Down	0000696/C00073	Incorporates into polypeptide chains and used in the production of α-ketobutyrate and cysteine *via S*-adenosylmethionine. Transulfuration reactions produce cysteine from homocysteine and serine also produces α-ketobutyrate, the latter being converted first to propionyl-CoA and then *via* a three-step process to succinyl-CoA
Glutathione	3.30 (dd); 2.96 (dd)	Up	0000125/C00051	An antioxidant and a coenzyme in various enzymatic reactions. Found almost exclusively in its reduced form
(B) Glycolysis and TCA cycle				
Glucose	5.22 (d); 4.64 (d); 3.89 (dd); 3.83 (m); 3.73 (m); 3.52 (dd); 3.46 (m); 3.40 (td); 3.23 (dd)	Down	0000122/C00031	Primary source of energy
Pyruvate	2.46 (s)	Down	0000243/C00022	Used to construct alanine and be converted into ethanol. Supplies energy to living cells through the TCA cycle when oxygen is present (aerobic respiration) and alternatively ferments to produce lactate in the absence of oxygen
Oxaloacetic acid	2.38 (s)	Down	0000223/C00036	Intermediate in the TCA cycle and converted to aspartic acid by a transamination reaction
Lactate	4.11 (dd); 1.32 (d)	UP	0000190/C00186	Alternative by-product in anaerobic respiration
Citrate	2.64 (d); 2.52 (d)	Down	0000094/C00158	Component of the TCA cycle
Succinate	2.39 (s)	Down	0000254/C00042	Component of the TCA cycle
Fumarate	6.4 (s)	Down	0000134/C00122	Intermediate in the TCA cycle
(C) Organic acids				
2-Hydroxybutyric acid	3.99 (dd); 1.73 (m); 1.64 (m); 0.88 (t)	Down	0000008/C05984	Catabolize L-threonine or synthesize glutathione
Acetone	2.22 (s)	Up	0001659/C00207	
Methylmalonic acid	3.16 (m); 1.23 (d)	Down	0000202/C02170	Methylmalonate is a malonic acid derivative that is an intermediate in the metabolism of fat and protein
Acetate	1.91 (s)	Down	0000042/C00033	Used by organisms in the form of acetyl-coA
Malonate	3.11 (s)	Down	0000691/C00383	Malonic acid (propanedioic acid) is a dicarboxylic acid. The ionized form of malonic acid and its esters and salts are known as malonates. Malonic acid is the archetypal example of a competitive inhibitor: it acts against succinate dehydrogenase (complex II) in the respiratory electron transport chain
α-Keto isovaleric acid	3.01(dd); 1.09 (d)	Down	0000019/C00141	A branched chain organic acid and a precursor to leucine and valine synthesis. A degradation product from valine and the starting compound for R-pantothenate (vitamin B5) biosynthesis pathway
3-Hydroxybutyric acid	4.16 (m); 2.41 (m); 2.31 (m); 1.06 (d)	Up	0000357/C01089	Also β-hydroxybutyrate. A ketone body, whose important function is to provide acetoacetyl-CoA and acetyl-CoA for synthesis of cholesterol, fatty acids, and complex lipids
p-Hydroxyphenylacetate	7.15 (d); 6.85 (d); 3.44 (s)	Down	0000020/C00642	
2-Hydroxyisovaleric acid	3.84 (d); 2.01 (m); 0.95 (d); 0.82 (d)	Up	0000407	Intermediate product of the catabolism of valine to isobutyl alcohol
(D) Nucleotide metabolism and urea cycle				
Formate	8.44 (s)	Down	0000142/C00058	Source of one-carbon groups for the synthesis of 10-formyl-THF and other one-carbon intermediates. Primarily used for purine synthesis and thymidylate synthesis
Uracil	7.53 (d); 5.79 (d)	Down	0000300/C00106	Involved in pyrimidine and β-alanine metabolism. Associated with pantothenate and CoA biosynthesis
Hypoxanthine/adenine	8.18 (s); 8.20 (s)	Down	0000157/C00262	Naturally occurring purine derivative and a reaction intermediate in the metabolism of adenosine and formation of nucleic acids by the salvage pathway. Also a spontaneous deamination product of adenine
UTP	7.95 (d)	Down	0000285/C00075	Important extracellular signaling molecule. Principally serves as a substrate for the synthesis of RNA during transcription
NAD^+^	9.33 (s); 9.15 (d); 8.83 (d); 8.42 (s); 8.19 (m); 6.13 (d); 6.08 (d); 6.02 (d)	Down	0000902/C00003	Coenzyme in redox reactions
ATP	8.6 (s); 8.17 (s); 6.14 (d); 8.53 (s); 8.26 (s)	Down	0000538/C00002	Maintains cellular energy homeostasis as well as signal transduction as cyclic AMP
Xanthine	7.89 (s)	Down	0000292/C00385	Product of the pathway of purine degradation
Ornithine	1.65–2.00 (mm); 3.04 (t)	Up	0000214/C00077	Non-proteinogenic amino acid that plays a role in the urea cycle. Also a precursor of citrulline and arginine
Urea	5.78 (s)	Up	0000294/C00086	Highly soluble organic compound formed by the deamination of amino acids. Principal end-product of protein catabolism
(E) Lipid metabolism				
Carnitine	3.19 (s)	Down	0000062/C00318	Ubiquitous compound biosynthesized from the amino acids lysine and methionine and involved in the transport of long-chain fatty acids. Fatty acids are broken down to acetyl-CoA through β-oxidation, which in yeast takes place exclusively in peroxisomes. Acetyl-CoA is then used in the glyoxylate cycle for gluconeogenesis and formation of carbohydrates or transported to the mitochondrion for the generation of metabolic energy through the TCA cycle
Ethanol	3.65 (q); 1.71 (t)	Up	0000108/C00469	Involved in glycolysis/gluconeogenesis and pyruvate metabolism
Phosphorylcholine	4.16 (dd); 3.59 (t); 3.21 (s)	Up	0001565/C00588	Intermediate in the choline and phosphatidylcholine biosynthesis pathway. Phosphatidylcholine is a phospholipid, which is the major structural component of biological membranes
Ethanolamine	3.81 (dd); 3.13 (dd)	Up	0000149/C00189	Important head group for phospholipids, which are the major structural components of biological membranes. Precursor in the phosphatidylethanolamine biosynthesis pathway. Also used for the biosynthesis of choline, which is another important head group for phospholipids
Choline	4.06 (dd); 3.5 (dd); 3.18 (s)	Down	0000097/C00114	Important head group for phospholipids, which are the major structural components of biological membranes. Precursor in the phosphatidylcholine biosynthesis pathway
Glycolate	3.94 (s)	Down	0000115/C00160	
Glycerol	3.77 (m); 3.65 (dd); 3.55 (dd)	Up	0000131/C00116	Important component of triglycerides and phospholipids
Myoinositol	4.05 (t); 3.61 (t); 3.52 (dd); 3.26 (t)	Down	0000211/C00137	Plays an important role as the structural basis for a number of secondary messengers in eukaryotic cells, including inositol phosphates, phosphatidylinositol, and phosphatidylinositol phosphate lipids

### Do AgNPs Cause Alteration in Histone Epigenetic Marks?

**Figure 7:**
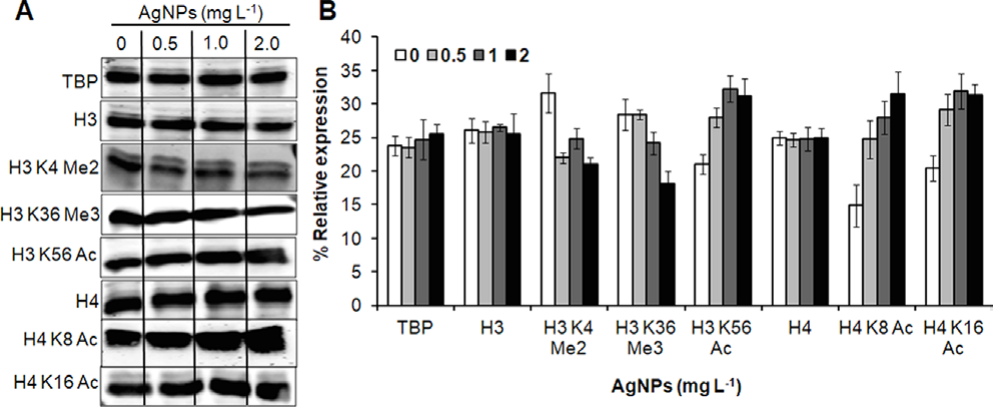
AgNPs cause alteration in histone PTMs. **(A)** Whole-cell protein extracts of untreated (0) and AgNP-treated yeast cells for 3 h at 30°C were analyzed for histone methylation (Me) and acetylation (Ac) marks by immunoblotting using the indicated antibodies. Anti-tata binding protein (Tbp), anti-histone H3, and anti histone H4 antibodies were used as loading control. **(B)** Densitometric analysis was performed by measuring the density of bands using the Alpha-Imager^©^ software and the percent relative expression (control vs. AgNPs) of the indicated histone modification was quantified. Data are mean ± SEM of three independent experiments.

To investigate the role of histone modification in cellular responses upon AgNP treatments, histone PTMs was analyzed by immunoblotting. A significant down-regulation in histone methylation (H3K4Me2 and H3K36Me3) was observed, whereas selected acetylation (H3K56Ac, H4K8Ac, and H4K16Ac) was up-regulated upon AgNP exposure (**Figure 7**).

## Discussion

A number of studies were reported previously to describe the green/biological methods of AgNP synthesis using a variety of plant and microbial cell extracts, including their toxic effects on different microbes, tumor cell lines, and mice models (McShan et al., 2014; Singh et al., 2014; Vazquez-Muñoz et al., 2017; Siddiqi et al., 2018). Here, we devised a new green (biological) method using gooseberry extract. Gooseberry was employed due to its very high amounts of antioxidants and polyphenolic compounds, thus acting as a strong reducing agent for AgNO_3_ and eventually providing stability to the particles (Rosarin et al., 2013; El-Sheekh and El-Kassas, 2014). It can also be seen that the absorption peaks were sharp, indicating the narrow size distribution of the nanoparticles. The recorded XRD pattern of synthesized AgNPs exhibits peaks at angles (2θ) 37.9°, 44.1°, 64.3°, and 77.3° corresponding to (111), (200), (220), and (311) reflection planes of silver lattice, respectively. In UV-vis spectra, it is quite obvious that there is a blue shift in absorption maxima, when gooseberry extract is reduced from 1 to 0.5 in ratio. The enhanced intensity and observed blue shift in the absorption peak suggest the decrease in the particle size of AgNPs. There are no extra peaks related to the by-products, which indicate the effectiveness of our synthesis method to fabricate the desired nanoparticles. TEM and SAED patterns further confirm the formation of AgNPs with supporting data of particle size distribution and dynamic light scattering (DLS). Our observations clearly suggest that the particles have no specific shapes, and average particle size ranges from approximately 50 to 70 nm.

After the successful synthesis and characterization of AgNPs, we investigated the effects of these AgNPs on the yeast *S. cerevisiae* to elucidate the mechanism(s) of toxicity in eukaryotes. Instead of rich growing media, sterilized DI water was used for AgNP treatment toward yeast cells. It was used to avoid the aggregation and accumulation of AgNPs as discussed previously (Babele et al., 2018; Babele, 2019). Preliminary results showed the inhibitory effect of AgNPs on the growth and survival of yeast cells as evidenced by CFU and spot assay in a dose-dependent manner. Furthermore, it was investigated whether the inhibition of cell growth was associated with cell membrane damage. PI and DCFDA staining revealed that AgNPs cause a remarkable increase in PI-positive cells and ROS accumulation, which indicate damage to the cell membrane and consequently cell death. Our results are in accordance to earlier reports, establishing that AgNPs cause several cellular responses, including ROS generation and cell death (McShan et al., 2014; Márquez et al., 2018).

Microorganisms that have evolved adaptive/stress responses were studied by adjusting metabolism through the modulation of gene expression, protein/enzyme level activity, and cellular concentration of metabolites, allowing them to survive under stressful and in ever-fluctuating environmental conditions (Pereira et al., 2018). Deregulation in the complete cellular metabolome and investigation of transcriptome and histone PTMs in response to AgNPs are the major objectives of this study. A significant deregulation in the key metabolites of the cells involved in various metabolic pathways has been observed. It has also been observed that almost all amino acids annotated in this study are down-regulated, except glutathione, glutamine, and cysteine. These observations indicate that AgNP exposure severely affects amino acid metabolism in yeast cells. The levels of reduced glutathione are increased, which is considered as a natural defense system of cells to cope with oxidative stress, as it plays an essential role in maintaining the intracellular redox environment for metal stress-generated inflammation and oxidative stress (Hatem et al., 2014; Ilyas and Rehman, 2015). The observed diminished level of glycine, a well-known precursor amino acid of glutathione biosynthesis, might be due to its utilization to replenish glutathione consumed by the cells (Ribas et al., 2014).

It is well established that the mitochondria (powerhouse of the cell) are fundamental bioenergetic organelles and fulfill the energy requirements of the cell. They participate in central carbon metabolism (CCM) and various other metabolic reactions of the cell, including stress mitigation, by providing precursors and intermediates for many other primary and secondary metabolites, including amino acids (Edirisinghe et al., 2016). The CCM and other metabolic pathways can be altered effectively in *S. cerevisiae* under stressed conditions (Wiebe et al., 2007; Guo et al., 2016). Metabolites identified in the present work show that metabolites linked to glycolysis, tricarboxylic acid (TCA) cycle, and pentose phosphate pathway, such as glucose, pyruvic acid, succinate, fumaric acid, citrate, and oxaloacetic acid, are drastically down-regulated, indicating that AgNPs affect the CCM. Earlier, an NMR based metabolomic study suggested that Ag^+^ and AgNPs significantly down-regulate the metabolites (citrate, succinate, and α-ketoglutarate) of glycolysis and Kreb’s (TCA) cycle in human skin keratinocytes (HaCaT cells; Carrola et al., 2016). Also, in an *in vivo* metabolic study, it appeared that the TCA cycle was down-regulated in the lungs of AgNP-injected mice (Jarak et al., 2017). Succinic acid, an indispensable metabolite in the TCA cycle, participates in the GABA pathway involved in the mitigation of ROS-mediated oxidative stress (Cao et al., 2013). The up-regulation of lactate, but down-regulation in the levels of glucose, manifests as a change into lactate or acetate to provide bounteous energy for survival. These results might encompass the imbalance in the aerobic metabolism and the TCA cycle. Moreover, acetate is also regarded as an expedient source of energy for stressed cells (Jiang et al., 2013). These observations are in consistent with the earlier reports in which nanoparticles cause oxidative stress and defects in mitochondrial and endoplasmic reticulum (ER) enzymes (Teodoro et al., 2011; Jarak et al., 2017). In aerobic metabolism, the formation of ROS is a natural by-product, but in excess they can chemically modify proteins and lipids by lipid peroxidation and oxidative stress, thus leading to a damage to vital organelles such as mitochondria, ER, and lysosomes (McShan et al., 2014; Márquez et al., 2018).

A drastic down-regulation in a number of organic acids such as malonate, α-keto isovaleric acid, p-hydroxyphenyl acetate, 2-hydroxybutyric acid, and methyl-malonic acid was also observed, whereas the concentrations of 2-hydroxyisovaleric acid, 3-hydroxybutyric acid, and acetone were up-regulated. These observations suggest that yeast cells modulate their metabolism to improve their viability under AgNP stress, as 3-hydroxybutyrate, one of the end products of fatty acid β-oxidation, functions as an alternative energy source (Newman and Verdin, 2014). Similar to our finding, there are several other metal oxide toxicity studies that have reported the increase in 3-hydroxybutyrate in the serum or urine, whereas the glucose level is found to be limited or the TCA cycle is impaired (Lei et al., 2008; Bu et al., 2010; Yan et al., 2012). In addition, it was proposed that the increased level of 3-hydroxybutyrate uses as an auxiliary energy source to replenish the energetic demands during tissue damage (Fabisiak et al., 2011; Lee et al., 2016).

AgNP exposure also affects the levels of many metabolites associated with nucleotide metabolism and urea cycle. These metabolites known to be involved in energy generation/transfer mechanisms, particularly uracil, UTP, hypoxanthine, NAD, and ATP, are down-regulated after treatment compared to control cells. Nucleotides are considered to play a vital role in diverse cellular processes. Moreover, nucleotides are assumed to be assimilated in various cofactors (e.g., NAD and coenzyme A) and also serve as precursors (e.g., UDP-glucose and GDP-mannose). A down-regulation in the level of formate was also observed, it is the only non-tetrahydrofolate (THF)-linked intermediate in one-carbon metabolism and produced from various cellular metabolic reactions. Formate is regarded as an important substitute to single-carbon source for *N* ^5^, *N* ^10^-methylene-THF generation; these are primarily used for purine and thymidylate biosynthesis and required for epigenetic methylation reactions of methyl groups for synthetic, regulatory mechanism (Brosnan and Brosnan, 2016). Recently, it was observed that this acid also has potential to combat against oxidative stress (Thomas et al., 2016).

Increment in the metabolites involved in lipid metabolism, i.e., glycerol, choline, ethanol, myo-inositol, and carnitine, are found in their levels. Glycerol is a well-known protective agent synthesized by yeast cells for environmental stress and maintains redox homeostasis (Paget et al., 2014). The increment in glycerol biosynthesis rate primarily signifies the alteration in stress defense of cells for AgNP toxicity. Choline and phosphorylcholine are also important metabolites that are found up-regulated. Glycerophosphocholine is also an important ingredient in eukaryotic cellular membranes. These are primarily convoluted in the membrane choline phospholipid metabolism pathway, in which phosphatidylcholine may be converted to glycerophosphocholine and further reconverted to phosphatidylcholine (Hu et al., 2008; Van Meer et al., 2008). As a result of nanotoxicity, cells consume glycerophosphocholine to generate phosphatidylcholine, which helps in maintaining the integrity of the cell membrane. This provides a path to propose a plausible mechanism of membrane component maintenance upon AgNP exposure. The results/findings of the present study are analogous and parallel to the results of a research conducted on human hepatoma (HepG2) cells exposed to citrate-coated AgNPs (Carrola et al., 2018).

Histone PTMs regulate the transcription and other DNA-dependent functions of eukaryotic cells and these PTMs are driven by specific histone-modifying enzymes. Cellular metabolites serve as co-substrates or enzyme inhibitors essentially required for proper enzyme activities. Therefore, cell metabolism can be influenced through histone modification dealing with local concentrations of metabolites (Fan et al., 2015). The epigenetic changes in response to cellular metabolism are vital for stress adaption and cell survival physiologically. There are numerous environmental factors that alter gene expression through covalent histone modifications and fundamental genome functions (Kouzarides, 2007; Fang et al., 2014). These modifications have not been much studied in response to biologically synthesized AgNPs. Some reports described that the sublethal dose of AgNP induces a drastic fall in hemoglobin levels in mouse erythroleukemia cells, imprinting diminished levels of H3K4me3 and H3K79me1 methylation. These results have been attributed to the direct binding of AgNPs to histone (H3/H4) proteins (Qian et al., 2015). A study on human cells reported the phosphorylation of histone H3 at serine 10 (p-H3S10) and its usefulness as a marker to scale the toxicity induced by AgNPs (Zhao and Ibuki, 2015). Here, we found significant changes in the methylation and acetylation marks of histone H3 and H4 proteins upon AgNP exposure, suggesting that either AgNPs induced metabolic alteration or their direct binding to nucleosomes may be involved in the histone modification.

## Conclusion

The present study describes the potential of ^1^H-NMR-based metabolomics in nanotoxicology. This approach is successfully used for the rapid and untargeted screening of biochemical modulations in *S. cerevisiae* upon AgNP exposure. The combined metabolomic and epigenetic alterations demonstrate that a very low concentration of biologically synthesized AgNPs has potential to induce nanotoxicity by modulating the metabolic machinery of yeast cells.

## Data Availability

The raw data supporting the conclusions of this manuscript will be made available by the authors, without undue reservation, to any qualified researcher.

## Author Contributions

PB conceived the whole idea, designed and performed the metabolic toxicity experiments, ASi performed and wrote the nanoparticle synthesis part. ASr helped in writing and correction of the MS.

## Funding

This work was supported by a project grant sanctioned to PB in the form of a national postdoctoral fellowship by SERB-DST, New Delhi, India (File No. PDF/000200).

## Conflict of Interest Statement

The authors declare that the research was conducted in the absence of any commercial or financial relationships that could be construed as a potential conflict of interest.
