# Supplementary material for: Bio-Inspired Silver Nanoparticles Impose Metabolic and Epigenetic Toxicity to Saccharomyces cerevisiae
Source: Front Pharmacol. 2019 Sep 12;10:1016. doi: 10.3389/fphar.2019.01016 (PMC6751407; doi:10.3389/fphar.2019.01016)
Supplement: Supplementary file 1 [file DataSheet_1.pdf]

Supplemantary information: Bio-inspired silver nanoparticles impose metabolic and epigenetic toxicity to *Saccharomyces cerevisiae*

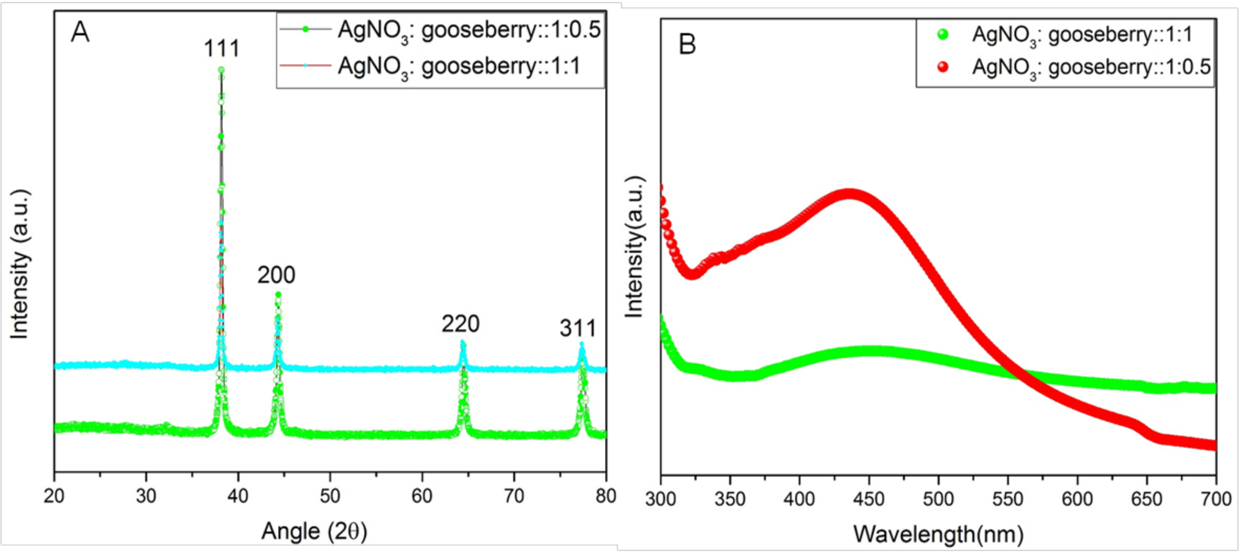

Fig. S1. Synthesis and characterization of AgNPs (A) XRD patterns and (B) UV-Visible spectra of AgNPs synthesized using gooseberry extract in different ratios.

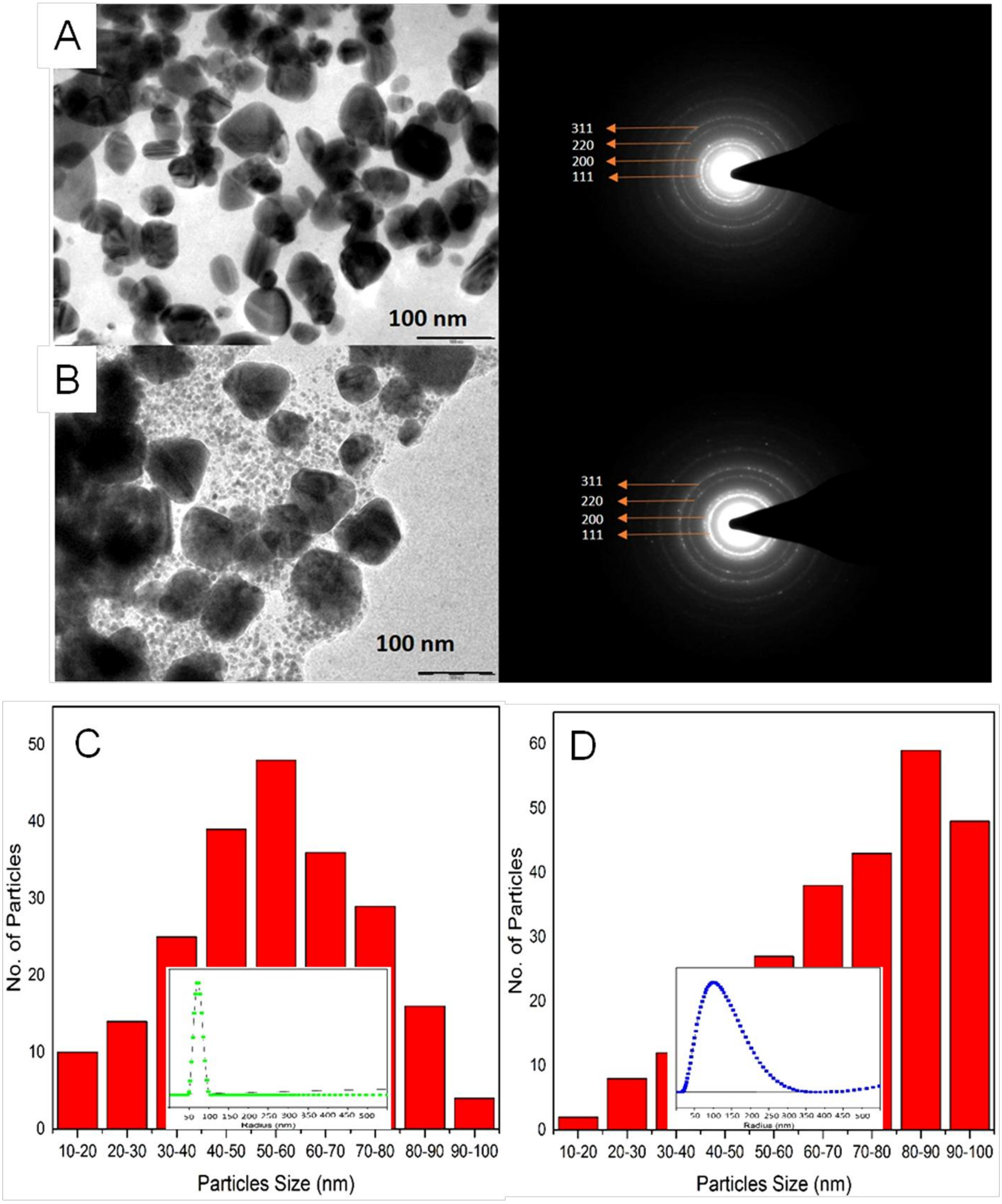

Fig. S2. Size distribution of AgNPs (A) and (B) are TEM and SAED patterns of AgNPs synthesized using $AgNO_3$ and gooseberry extract in 1:0.5 and 1:1 respectively. (C) and (D) are respective statistical particles sized distribution of AgNPs. Inset of Fig 2(C) and (D) are DLS data of AgNPs synthesized with 1:0.5 and 1:1 respectively.

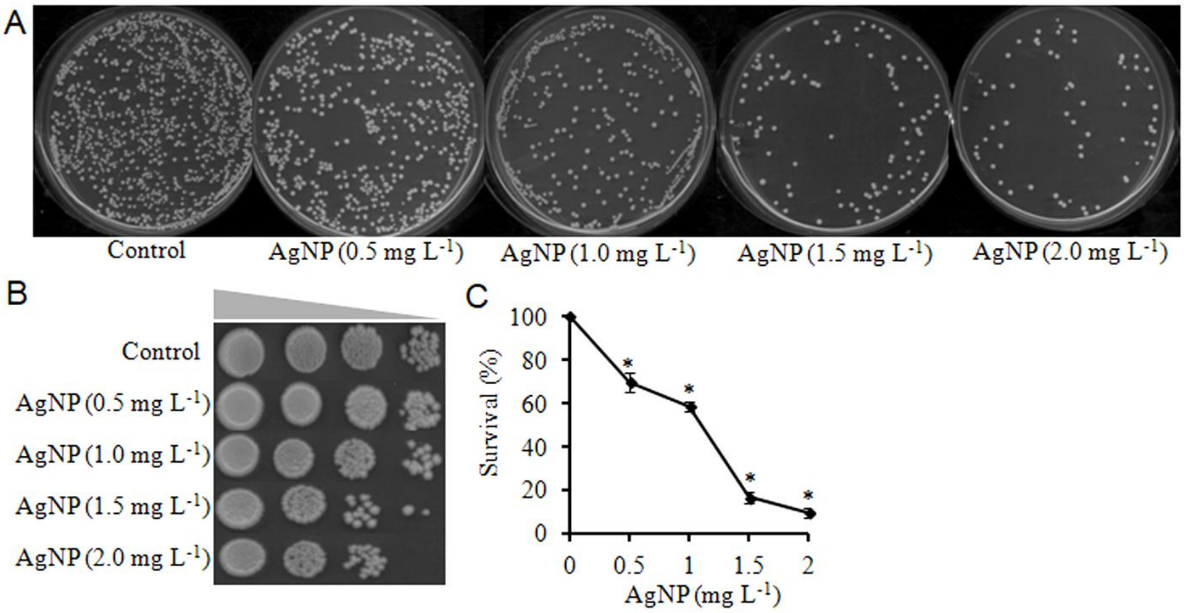

Fig. S3. Growth inhibition and cytotoxicity test of the biologically synthesized AgNPs to *S. cerevisiae* (A) The wild type yeast (BY4741) cells were co-incubated with different concentrations of AgNPs (mg L^{-1}) for 3 h and after plating on YPD-agar plate CFU were counted after 72 h of incubation (B) The wild type control and treated cells were spotted after ten-fold serially dilution and imaged after 72h (C) The percent of survival in each group was calculated as the cell number of each group divided by that of the control group X 100. The error bars indicate the standard deviations (n=3).). * indicates a significant difference between the treatment and the control groups ($P < 0.05$).

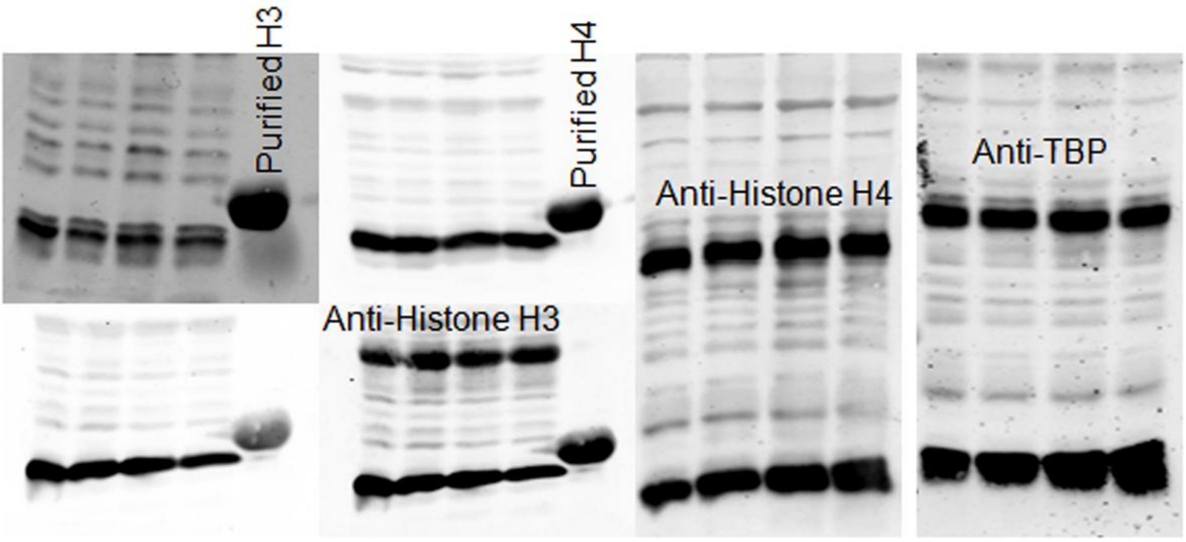

Fig. S4. Western blots showing the modifications in selected histone marks. Purified histone H3 and H4 proteins (shown in the last well of the blots) are used as marker to determine the size of histone proteins. Same blots are probed with anti-TBP, H3, and H4 antibodies these are used as loading control (as shown on the blots).

Supplementary Table 1: List of primers and genes information used in this study.

S. No.	Gene	Oligo Sequences	Description
1	ACT1	F-CCTTCTGTTTTGGGTTTGGA<br>R-CGGTGATTTCCTTTTGCATT	Actin; structural protein involved in cell polarization, endocytosis, and other cytoskeletal functions.
2	CRD1	F-TACGGCCTGAAAACCATTGC<br>R-CATGCCACACGACCAGGATA	Cardiolipin synthase; produces cardiolipin, which is a phospholipid of the mitochondrial inner membrane that is required for normal mitochondrial membrane potential and function and for correct integration of membrane-multispanning proteins into the mitochondrial outer membrane; required to maintain tubular mitochondrial morphology and functions in mitochondrial fusion; also required for normal vacuolar ion homeostasis.
3	PSD1	F-TACCGCTGAATGCGATGTCT<br>R-AACGTCTTCGCCTTGTGCTA	Phosphatidylserine decarboxylase; involved in phosphatidylcholine biosynthesis, positive regulation of protein processing, integral component of mitochondrial inner membrane; regulates mitochondrial fusion and morphology by affecting lipid mixing in the mitochondrial membrane.

4	ACO1	F-ATGTTATGGCAGGTCGTCCA R-ACCCATACCAGTAGCGGAGA	Aconitase; required for the tricarboxylic acid (TCA) cycle and also independently required for mitochondrial genome maintenance; component of the mitochondrial nucleoid; mutation leads to glutamate auxotrophy; human homolog ACO2 can complement yeast null mutant
5	SOD1	F-GTGTCTCTGCTGGTCCTCAC R-GGATAACGACGCTTCTGCCT	SuperOxide Dismutase- Cytosolic copper-zinc superoxide dismutase; detoxifies superoxide; stabilizes Yck1p and Yck2p kinases in glucose to repress respiration; phosphorylated by Dun1p, enters nucleus under oxidative stress to promote transcription of stress response genes; localized to the nucleus, cytosol, and mitochondrial intermembrane space.
6	SOD2	F-GCATTACACCAAGCACCATC R-GAGCCAGGTTTTCCCAGAAT	Superoxide dismutase- Mitochondrial manganese superoxide dismutase; protects cells against oxygen toxicity and oxidative stress; human mitochondrial SOD2 can complement a yeast null mutant and human cytoplasmic SOD1 can also complement when targeted to the mitochondrial matrix.
7	INO2	F-AACACACGAGCTCGGCATAA R-CTCAACAACCCCGAACTGGA	INOsitol requiring- Transcription factor; component of the heteromeric Ino2p/Ino4p basic helix-loop-helix transcription activator that binds inositol/choline-responsive elements (ICREs), required for derepression of phospholipid biosynthetic genes in response to inositol depletion; involved in diauxic shift.
8	INO4	F-CAACCCCAGGAAAGTCGGTC R-CCATCACCCAGCTCCCAAAT	Ionisitol requiring- Transcription factor involved in phospholipid synthesis; required for derepression of inositol-choline-regulated genes involved in phospholipid synthesis; forms a complex, with Ino2p, that binds the inositol-choline-responsive element through a basic helix-loop-helix domain.
9	CHO2	F-CGCATCGCGTGGTTAAAGAT R- CGGACACCAGAAGAACCTTGT	CHOline requiring- Phosphatidylethanolamine methyltransferase (PEMT); catalyzes the first step in the conversion of phosphatidylethanolamine to phosphatidylcholine during the methylation pathway of phosphatidylcholine biosynthesis.
10	YAP1	F-TACACGTGATGGCGAGGATA R-CCACTTCATTTTGCTGCTGA	Yeast AP-1- Basic leucine zipper (bZIP) transcription factor; required for oxidative stress tolerance; activated by H_2O_2 through the multistep formation of disulfide bonds and transit from the cytoplasm to the nucleus; Yap1p is degraded in the nucleus after the oxidative stress has passed; relative distribution to the nucleus increases upon DNA replication stress.
11	KGD1	F-GGTTAACTGCTGCCTGGGAA R-CCCAATCGATGCCTTCACCT	Alpha-KetoGlutarate Dehydrogenase- Subunit of the mitochondrial alpha-ketoglutarate dehydrogenase complex; catalyzes a key step in the tricarboxylic acid (TCA) cycle, the oxidative decarboxylation of alpha-ketoglutarate to form succinyl-CoA.
